# HD-03/ES: A Herbal Medicine Inhibits Hepatitis B Surface Antigen Secretion in Transfected Human Hepatocarcinoma PLC/PRF/5 Cells

**DOI:** 10.1155/2013/125398

**Published:** 2013-04-10

**Authors:** Sandeep R. Varma, R. Sundaram, S. Gopumadhavan, Satyakumar Vidyashankar, Pralhad S. Patki

**Affiliations:** Research and Development, The Himalaya Drug Company, Bangalore 562 123, India

## Abstract

HD-03/ES is a herbal formulation used for the treatment of hepatitis B. However, the molecular mechanism involved in the antihepatitis B (HBV) activity of this drug has not been studied using *in vitro* models. The effect of HD-03/ES on hepatitis B surface antigen (HBsAg) secretion and its gene expression was studied in transfected human hepatocarcinoma PLC/PRF/5 cells. The anti-HBV activity was tested based on the inhibition of HBsAg secretion into the culture media, as detected by HBsAg-specific antibody-mediated enzyme assay (ELISA) at concentrations ranging from 125 to 1000 **μ**g/mL. The effect of HD-03/ES on HBsAg gene expression was analyzed using semiquantitative multiplex RT-PCR by employing specific primers. The results showed that HD-03/ES suppressed HBsAg production with an IC_50_ of 380 **μ**g/mL in PLC/PRF/5 cells for a period of 24 h. HD-03/ES downregulated HBsAg gene expression in PLC/PRF/5 cells. In conclusion, HD-03/ES exhibits strong anti-HBV properties by inhibiting the secretion of hepatitis B surface antigen in PLC/PRF/5 cells, and this action is targeted at the transcription level. Thus, HD-03/ES could be beneficial in the treatment of acute and chronic hepatitis B infections.

## 1. Introduction

Hepatitis B virus (HBV) infection is a major health problem throughout the world, affecting more than 350 million people who are carriers of this virus that can cause chronic hepatitis, liver cirrhosis, and hepatocellular carcinoma [1]. A variety of serological markers appear following the infection with HBV, and first among these is HBsAg (hepatitis B surface antigen), which is observed two to three weeks before the clinical and biological symptoms appear. Prevalence of HBsAg in India varies from 1 to 13 percent with an average of 4.7 percent [2–4]. The molecular diagnosis which detects the HBsAg in the serum samples plays a significant role in the early diagnosis during hepatitis B (HB) infection.

PLC/PRF/5 is a continuous human hepatocarcinoma cell line whose genome contains integrated HBV DNA and secretes two of the hepatitis B virus envelope proteins [5]. The cells could secrete HBsAg continuously into the culture medium [6, 7]. These cells are suitable to study the effects of drugs on HBsAg expression and secretion [6]. Since the cells do not produce infectious virion particles, it is safe to handle the cell line with biosafety level 2 containment [8]. 

Several antivirals are currently available for the treatment of HBV, which include IFN-*α*, lamivudine, entecavir, telbivudine, and tenofovir. However, interferon therapy has limited efficacy, is slow-acting, and frequently causes adverse effects [9]. Interferon therapy is effective only for about 30 to 40 percent of the patients with chronic HBV infection. Undesirable side effects of interferon treatment are found such as fever, malaise, fatigue, depression, hair loss, neutropenia, and thrombocytopenia [10]. Lamivudine also produces response in a modest proportion of patients and causes a few side effects [11]. Moreover, antiviral drugs and interferon are expensive.

Herbal compounds from plant origin are leading for new drug discovery for infectious and noninfectious diseases. Several hundred plant species have been reported to possess antiviral properties and some have been utilized to treat patients [12]. HD-03/ES is a novel herbal formulation used for the treatment of HBV infections and is marketed as Liv.52 HB. HD-03/ES is a capsule formulation consisting of 125 mg each of hydroalcoholic extracts of the roots of herbs, *Cyperus rotundus* and *Cyperus scariosus*. The anti-HBV activity of HD-03/ES has been reported by several workers [13–15]. However, the molecular mechanism behind the anti-HBV activity of HD-03/ES has not been studied well *in vitro*. The present study investigated the effect of HD-03/ES on the inhibition of HBsAg secretion and its gene expression in PLC/PRF/5 cells.

## 2. Materials and Methods

### 2.1. Materials

PLC/PRF/5 cells were obtained from National Center for Cell Science (NCCS), Pune. Dulbecco's modified Eagle's medium (DMEM), fetal bovine serum (FBS), 3-(4,5-dimethylthiazol-2-yl)-2,5-diphenyl tetrazolium bromide (MTT), TRI reagent, and custom-prepared oligonucleotides, were obtained from Sigma Chemical Co. (St Louis, MO, USA). HBsAg Ultra ELISA kit was purchased from Bio-Rad, France. Penicillin and streptomycin were from Hi-media, Mumbai, India. Moloney murine leukemia virus (MMLV) reverse transcriptase, dNTP, and Taq DNA polymerase were from MBI Fermentas (Glen Burnie, MD, USA). 

### 2.2. Extraction of HD-03/ES

HD-03/ES granules were obtained from the Formulation and Development Department, The Himalaya Drug Company, India. About 100 g of HD-03/ES granules was packed in a Soxhlet extraction apparatus. The material was extracted using methanol for 8 hours at 80°C. The extract was concentrated using a rotary evaporator. The dry residue was subjected to *in vitro* studies.

### 2.3. Cell Culture and Cytotoxicity Assay

PLC/PRF/5 cells were cultured in DMEM high glucose medium supplemented with 10% FBS, 100 IU penicillin, and 100 *μ*g streptomycin per mL at 37°C and 5% CO_2_. PLC/PRF/5 cells at density of 2 × 10^5^ mL were seeded in 96-well plates and incubated overnight at 37°C and 5% CO_2_. HD-03/ES was dissolved in 0.5% DMSO in DMEM high glucose medium and used for the experiments. The cells were treated with different concentrations of HD-03/ES in culture media and incubated for 24 h at 37°C and 5% CO_2_ to determine cytotoxicity of the extract. Cell control and vehicle control were also maintained. Cell viability was tested by MTT assay after exposing the cells to 1 mg/mL MTT for 3 h at 37°C. The blue formazan product was solubilised in DMSO and optical density measured at 540 nm [16]. Nontoxic concentrations of HD-03/ES were used for further experiments.

### 2.4. HBsAg Detection

PLC/PRF/5 cells at the concentration of 5 × 10^4^ mL were seeded in a 24-well plate and incubated overnight at 37°C. The cells were treated with four nontoxic concentrations of HD-03/ES and incubated for 24 h at 37°C and 5% CO_2_. At the end of the incubation period, the supernatant was collected by centrifugation at 1000 rpm for 10 min at 4°C. The supernatant was collected in a fresh 1.5 mL microfuge tube and stored at −20°C for ELISA. The cell pellet was stored at −80°C for RNA isolation. 

The diagnostic kit for HBsAg (ELISA) from BioRad, France, was used for the detection of HBsAg in the culture medium. The assay was carried out according to the manufacturer's protocol. The absorbance was measured at 450 nm for determining the HBsAg present in the samples. The percentage inhibition of HBV by HD-03/ES was calculated over the cell control. 

### 2.5. RNA Isolation and RT-PCR

Total RNA was isolated from control cells and HD-03/ES-treated cells using TRI reagent and the RNA was stored at −80°C. RNA was subjected to DNase I treatment (10 *μ*g DNase I for 5 min at 65°C and cooled in ice for 1 min). RNA was quantified using spectrophotometer and the quantity of RNA was determined. One microgram of RNA was reverse-transcribed using Oligo-dT Primer at 42°C as described by us earlier [17]. The cDNA was stored at −20°C for further PCR reactions. A semiquantitative multiplex PCR was designed to compare the RT-PCR products of S gene and pre-S gene with GAPDH gene products to determine the relative levels of expression of HBsAg. PCR was carried out to amplify the HBsAg (S and pre-S genes) using specific primers in the second-strand synthesis. The GAPDH primers were also added to the same tube for each PCR reaction. Since the annealing temperatures of HBsAg gene and GAPDH were similar (60°C), the annealing temperature of the reaction was fixed at 60°C. The primer sequence for S gene was 5′-CCCAATACCACATCATCC-3′ (sense) and 5′-GGATTGGGGACCCTGCGC-3′ (antisense). The primer sequences used for pre-S were 5′-GGGTCACCATATTCTTGG-3′ (sense) and 5′-GTCCTAGGAATCCTGATG-3′ (antisense). For GAPDH gene, the primer sequence used was 5′-ACCACAGTCCATGCCATCAC-3′ (sense) and 5′-TCCACCACCCTGTTGCTGTA-3′ (antisense). The PCR reaction was subjected to 36 seconds of denaturation at 95°C, followed by 25 cycles of denaturation at 95°C for 36 sec, annealing at 60°C for 30 sec and extension at 72°C for 90 sec. A final extension at 72°C for 10 min completed the PCR programme. The PCR products were analyzed on 2% agarose gel stained with ethidium bromide and photographed under exposure to UV light. A standard molecular weight marker was resolved along with the samples to differentiate the cDNA amplicons in the agarose gel. Densitometric analysis (Image J software, Rasband, USA) was carried out to find the differences in the expression of the selected genes. 

## 3. Statistical Analysis

Data were analyzed to determine mean ± SD. Statistical analysis of the data was done by Student's unpaired *t*-test using GraphPad Prism software, (San Diego, USA). *P* value of less than 0.05 was considered significant.

## 4. Results

**Figure 1:**
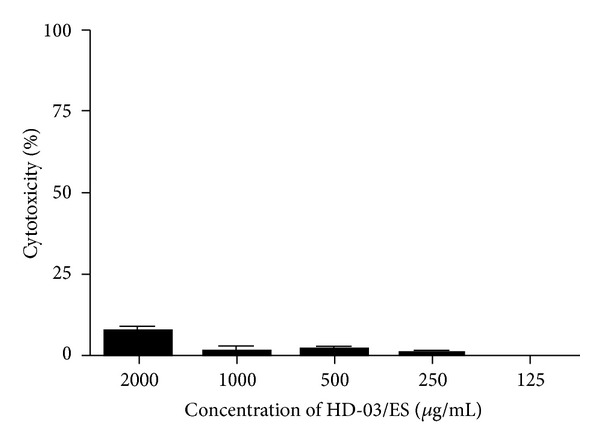
Cytotoxicity of HD-03/ES on PLC/PRF/5 cells. PLC/PRF/5 cells were incubated for 24 hr with different concentrations of HD-03/ES and the cell viability was then determined using an MTT assay. Data are expressed as percentage of control (*n* = 3).

**Figure 2:**
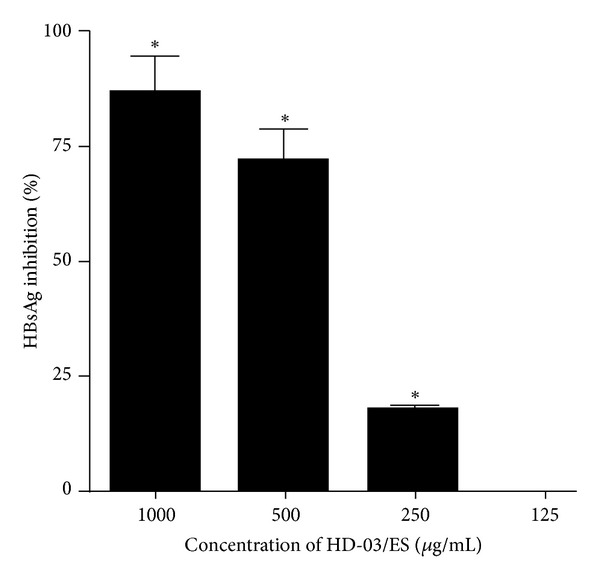
Effect of HD-03/ES on HBsAg secretion in PLC/PRF/5 cells by ELISA. PLC/PRF/5 cells were treated with four nontoxic concentrations of HD-03/ES for 24 hrs and the supernatant was assayed for ELISA using HBsAg Ultra ELISA kit. The percentage inhibition of HBsAg was calculated over control. Data is representative of three experiments. *P < 0.05.

Prior to the investigation of the anti-HBV effects, any putative cytotoxic effects of the HD-03/ES extract on PLC/PRF/5 cells were studied by MTT assay. The result of the cytotoxicity measurement of HD-03/ES extract on PLC/PRF/5 cells is shown in Figure 1. The percentage toxicity of HD-03/ES at 2000, 1000, 500, 250, and 125 *μ*g/mL was found to be 8.75, 1.25, 0.75, 0.25, and 0.00, respectively, in PLC/PRF/5 cells. The amount of HBsAg secreted into the cell culture medium was determined by ELISA. Four nontoxic concentrations (1000, 500, 250, and 125 *μ*g/mL) were used for HBsAg detection in PLC/PRF/5 cells. The optical density (OD) was read at 450 nm using an ELISA plate reader and the percentage of HBsAg secretion by the drug-treated cells was calculated over cell control. Each experiment was repeated three times and the results showed that HD-03/ES at concentrations of 1000, 500, and 250 *μ*g/mL inhibited HBsAg secretion by 86.38, 71.17, and 17.1%, respectively, as compared to the control (Figure 2). At 125 *μ*g/mL, HD-03/ES did not inhibit HBsAg in PLC/PRF/5 cells. However, at higher concentrations, HD-03/ES suppressed HBsAg production in PLC/PRF/5 cells with an IC_50_ of 380 *μ*g/mL.

**Figure 3:**
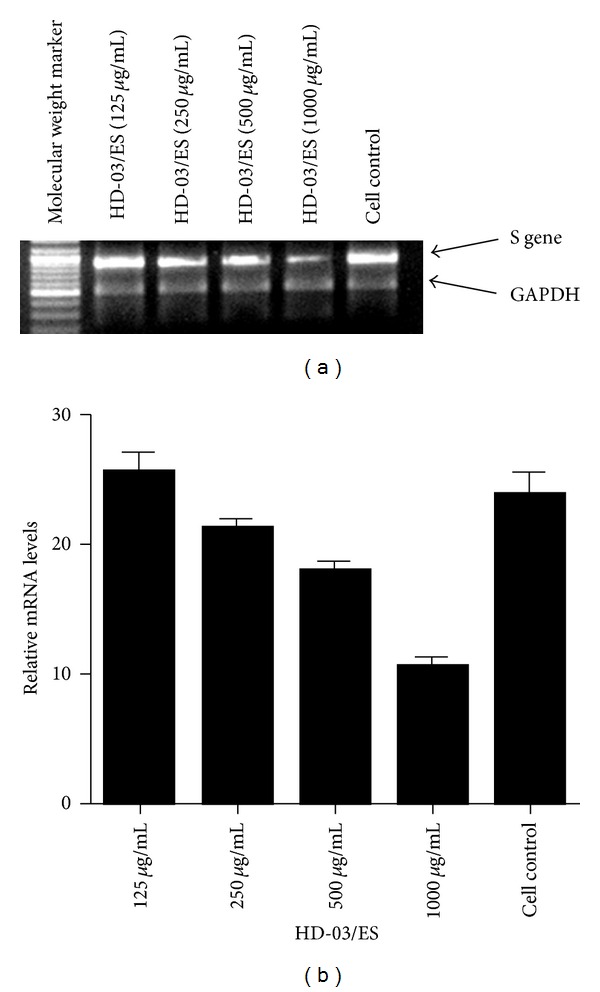
The effect of HD-03/ES on HBsAg gene expression. PLC/PRF/5 cells were treated with or without HD-03/ES at three nontoxic concentrations. RNA was isolated from drug-treated and -untreated cells and multiplex RT-PCR was performed using specific primers as described in the text. (a) RT-PCR product of S-gene and GAPDH resolved in 2% agarose gel. (b) Densitometric analysis of the gene transcripts and the values depict arbitrary units. Data is representative of two experiments.

**Figure 4:**
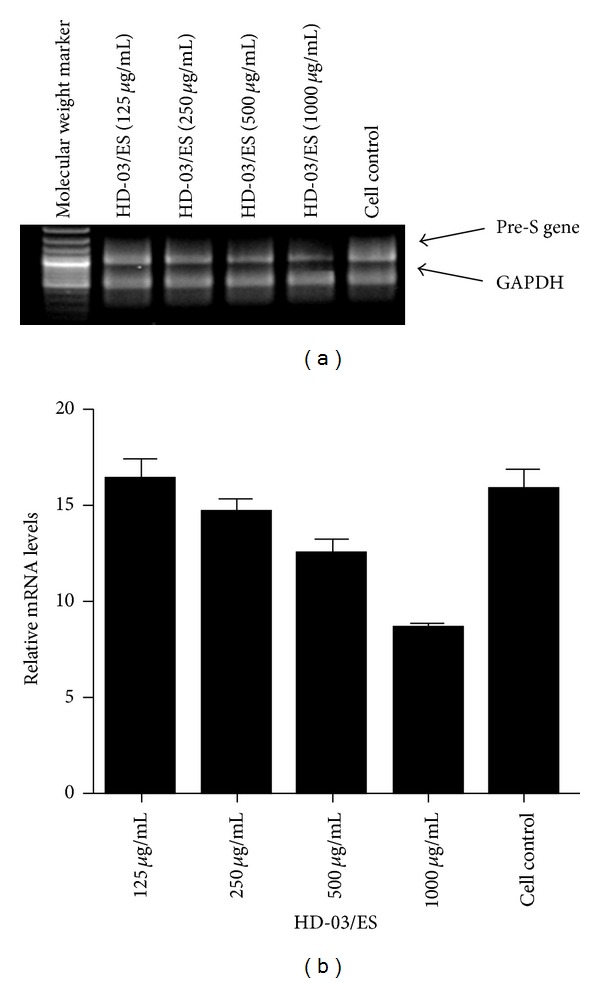
The effect of HD-03/ES on HBsAg gene expression. PLC/PRF/5 cells were treated with or without HD-03/ES at three nontoxic concentrations. RNA was isolated from drug-treated and -untreated cells and RT-PCR was performed using specific primers as described in the text ((a) RT-PCR product of pre-S gene and GAPDH resolved in 2% agarose gel; (b) densitometric analysis of the gene transcripts and the values depict arbitrary units). Data is representative of two experiments.

In order to check whether the inhibitory effect of HBsAg by HD-03/ES is targeted at the transcription level, semiquantitative multiplex RT-PCR was carried out using the RNA isolated from HD-03/ES-treated/untreated cells. Both S-gene- and pre-S-gene-specific primers were employed to amplify the gene encoding HBsAg in PLC/PRF/5 cells. The amplification yielded specific cDNAs corresponding to S gene (625 bp) and pre-S gene (553 bp) (Figures 3 and 4). Densitometric analysis compared the gene expression levels of the amplicons in comparison with GAPDH, the internal control. The results showed that HD-03/ES at 1000 and 500 *μ*g/mL suppressed the expression levels of HBsAg as compared to the cell control. The HBsAg expression levels in the cells treated with HD-03/ES extract were less than the control (Figures 3 and 4). The internal control, GAPDH, was uniformly amplified in all the samples. 

## 5. Discussion

Chronic HBV infection remains a major public health problem worldwide as well as a therapeutic challenge. Various treatments for chronic HBV infections have had only limited success [18]. The long-term effects of the recent advanced techniques employed to eliminate the virus, including therapy with nucleoside analogs and other virus-replication inhibitors [19], are yet to be determined. Since HBV reverse transcriptase lacks proof-reading function, the virus shows rapid mutagenesis thus creating a large number of variants, some of which show resistance to antiviral drugs. This phenomenon is responsible for the low efficacy of the current drugs and the high rates of drug resistance [20, 21]. Therefore, there is an urgent need to develop new anti-HBV drugs.

HD-03/ES is a herbal medicine used for curing hepatitis B and contains the extracts of *Cyperus rotundus* and *Cyperus scariosus* roots. Several clinical trials and *in vitro* studies have been carried out which confirmed the anti-HBV activity of HD-03/ES [13–15, 22]. A previous study reported that HD-03/ES inhibited alanine aminotransferase and HBV DNA [14]. A recent study on HD-03/ES showed that the drug at 5 and 2.5 mg/mL inhibited 1.5 pg/mL of the HBV virus, and the drug prevents HBV infection by possibly interfering with the viral entry [13]. However, the molecular mechanism behind the anti-HBV activity of HD-03/ES has not been studied well. Though previous studies have shown that HD-03/ES suppresses HBsAg, [13, 14] the effect of HD-03/ES on the HBsAg gene expression has not been studied *in vitro*. The present study investigated the cellular and molecular effects of HD-03/ES on the HBsAg using PLC/PRF/5 cells. 

Stable cell lines with integrated HBV genomes, namely, PLC/PRF/5 cells, are commonly used for assessing the action of drugs on HBsAg secretion [23, 24]. The property of PLC/PRF/5 cells to secrete HBsAg in the supernatant was used in the present study to evaluate the anti-HBV properties of HD-03/ES. HD-03/ES extract did not produce cytotoxic effect on PLC/PRF/5 cells within a reasonable dose range. It was seen that HD-03/ES at 1000 and 500 *μ*g/mL concentrations inhibited the secretion of HBsAg by 86.38 and 71.17%, respectively, for a period of 24 hours. However the lower concentrations were not successful in inhibiting the HBsAg in PLC/PRF/5 cells. The lack of cytotoxicity on PLC/PRF/5 cells, at the concentrations tested, indicates that the decrease in HBsAg is not due to an adverse effect of the drug on cell viability. In order to further confirm that the inhibition is HBsAg specific, the secretion of albumin in cell culture supernatants was checked (data not shown). The results showed that the albumin content in the cell supernatants of drug-treated/untreated cells was similar (data not shown). This study showed that the secretion of other cellular proteins like albumin was not altered by HD-03/ES at the doses tested. This result suggested that the antiviral effect of HD-03/ES might be more specific to the HBV.

PLC/PRF/5 cells contain six hepatitis B viral genomes integrated into the high molecular weight host DNA. The cells secrete only sAg and do not produce hepatitis B core antigen or free viral particles [25]. Both pre-S1 and pre-S2 proteins are expressed on the surface of HBsAg particles and are the essential components of complete virions and HBsAg filaments [26]. The expression of these envelope proteins originates from the HBV DNA coding for the respective genes, which is integrated into the cellular genome [23]. The pre-S2 mRNA encodes albumin receptors, which bind to pHSA and mediate viral attachment to the hepatocytes [27]. Pre-S1 and pre-S2 proteins are detectable in the serum of patients with acute and chronic hepatitis B virus infection when there are high levels of viral replication, and the clearance of these antigens from serum usually correlates with the prognosis of hepatitis B virus infection.

In order to assess whether the antiviral effect is due to the suppression of HBsAg gene, semiquantitative multiplex RT-PCR was carried out to amplify the regions coding the HBsAg gene in PLC/PRF/5 cells. The results showed that in drug-treated/control cells, HD-03/ES dose dependently suppressed the HBsAg gene expression. Densitometric analysis of the transcripts of S gene and Pre-S gene showed that HD-03/ES at higher concentrations, namely, 1000 and 500 *μ*g/mL, has downregulated the HBsAg gene expression in PLC/PRF/5 cells. Thus, it could be concluded that HD-03/ES inhibits HBV by inhibiting HBsAg at the transcription level.

This study suggests that in liver cells which have integrated HBV DNA, both S and pre-S antigen secretion could be inhibited by HD-03/ES. Since truncated pre-S antigen has been shown to be a transactivation protein possibly involved in the oncogenic process, the effect of HD-03/ES on pre-S gene is of therapeutic relevance. The impact of HD-03/ES on HBV inhibition by proteins, such as core and polymerase associated with full replication, deserves further study.

## 6. Conclusion

In conclusion, we have studied the *in vitro* anti-HBV effect of HD-03/ES in transfected human hepatocarcinoma cells. HD-03/ES suppressed HBsAg production with an IC_50_ of 380 *μ*g/mL in PLC/PRF/5 cells for a period of 24 h. HD-03/ES also downregulated HBsAg gene expression in PLC/PRF/5 cells. Previous reports have clearly indicated the anti-HBV activity of HD-03/ES. The main thrust of the present study is that, besides other methods of interference, HD-03/ES is capable of suppressing HBsAg, and the action is targeted at the transcription level.
